# Use of a Sorption Column with Polyurethane/Graphene Core Combined with an Electroflotation Reactor for Oily Wastewater Treatment

**DOI:** 10.3390/polym17081127

**Published:** 2025-04-21

**Authors:** Tiago Mari, Matheus V. G. Zimmermann, Bruna Rossi Fenner, Francisco Maciel Monticeli, Heitor Luiz Ornaghi Júnior, Camila Baldasso, Ademir J. Zattera

**Affiliations:** 1 Postgraduate in Process Engineering and Technologies Program, University of Caxias do Sul, Francisco Getúlio Vargas St., 1130, Caxias do Sul 95070-560, RS, Brazil; tmari1@ucs.br (T.M.); bruna.fenner@hotmail.com (B.R.F.); ornaghijr.heitor@gmail.com (H.L.O.J.); cbaldasso@ucs.br (C.B.); ajzatter@ucs.br (A.J.Z.); 2 Postgraduate Program in Materials Science and Engineering (PPGCEM), Universidade do Extremo Sul Catarinense (UNESC), Criciúma 88806-000, SC, Brazil; matheus.vgz@unesc.net; 3 Department of Aerospace Structures and Materials, Faculty of Aerospace Engineering, Delft University of Technology, 2629 HS Delft, The Netherlands

**Keywords:** PU foams, graphene, effluent treatment, electroflotation

## Abstract

Discharging oil-contaminated wastewater into the environment without adequate treatment can have a negative impact on water resources, public water and wastewater treatment systems, and even human health. In this sense, it is essential to develop compact, easily automated, low-cost, and highly efficient unitary treatment processes in order to comply with legal requirements regarding effluent emission standards for water bodies. Therefore, the present study consisted of the development of two treatment processes aimed at the separation of oil emulsions stabilised by anionic surfactants: a sorption column using polyurethane/graphene foam composites as sorbent material and a continuous flow AC electroflotation reactor. Initially, composites with 0.5% and 1% *w*/*w* graphene (based on polyol mass) were developed using a dispersing agent (1-methyl-2-pyrrolidone). The foams were characterised in terms of morphology and mechanical and sorption properties. In the fixed bed column, the foams retained up to 77.15% of the emulsified oil and 52.36% of the anionic surfactants. In the continuous flow electroflotation reactor, emulsified oil removal efficiencies above 90% were achieved at all electrical currents tested, and up to 88.6% of anionic surfactants were removed at an electrical current of 150 A. Given the advantages and disadvantages of the two oily effluent treatment processes, their combined use in the same system proved promising.

## 1. Introduction

Unregulated industrial wastewater is likely to be a significant source of unintentional releases of pollutants into the environment. Research shows that wastewater from various industrial activities is associated with adverse health effects, including cancer, immune dysfunction, and respiratory diseases [1]. The National Inspectorate for Environmental Protection (PIOS) has reported that approximately 60% of wastewater poses either a potential or actual risk to public health and environmental safety. To ensure effective and safe wastewater management, it is essential to carry out risk assessments that include hazard identification, exposure assessment and risk characterisation [2]. Industrial wastewater typically contains pathogenic bacteria such as E. coli and Salmonella, which can cause diseases such as cholera, typhoid, and various allergic reactions [3]. It is therefore important to develop highly efficient adsorbents to reduce the environmental impact, such as polyurethane foam [4].

Polyurethane (PU) foams can also be classified as 3D materials due to their high adsorption capacity, low density, high porosity, and mechanical flexibility. However, PU is mainly composed of polar and non-polar bonds, which do not have good selectivity when oil and water are presented [5,6]. Therefore, PU foam needs to have superhydrophobic and superoleophilic properties to reduce the surface energy and ensure an increase in surface rugosity at the nano/microscopic scale [7]. However, surface modification alone is not sufficient to promote efficient oil sorption. Other parameters such as capillary forces [8] and porous connectivity [9] are also important to ensure maximum oil sorption capability. Re-use of the foam after many cycles of sorption, saturation, and compaction is also critical for practical industrial application [10]. Preliminary studies have shown that these foams can be reused more than 100 times after the first adsorption cycle [11,12]. As mentioned above, polyurethane foam per se has a relatively low hydrophobicity. Therefore, the use of nanoparticles such as graphene can be used to modify the external and internal surfaces of the foam to make it superhydrophobic and superoleophilic [13].

Graphene is a relatively new class of materials that can be used in various fields [14,15,16]. In the field of wastewater oil treatment, many researchers have used graphene and its derivatives to develop superwetting materials for oil and water separation. Sel-vasembian et al. [17] presented recent advances in the preparation of polyurethane-based adsorbents for the removal of metals, dyes, pesticides, hydrocarbons, and emerging contaminants, presenting the main reaction mechanisms, kinetics, and synthesis, in addition to an in-depth analysis of the fate, behaviour, and health risks of spent PU adsorbents, removal of microbial contaminants, industrial application, and regeneration recycling of spent adsorbents. Among the polyurethane-based adsorbents, PU with graphene is mentioned. Xue et al. [4] studied the selective adsorption and recovery of precious metal ions from water and metallurgical slag by polymer brush graphene-polyurethane composite. The authors claimed that the adsorption process was influenced by factors such as solution pH, contact time, initial metal ion concentration, and temperature. The adsorption capacity of poly-Cys-g-PDA@GPUF exceeded that of many adsorbents documented in the literature. In addition, the influence of temperature on sorption was evaluated, and the thermodynamic parameters were determined, suggesting that the adsorption process is both exothermic and spontaneous. The adsorption behaviour of PM ions on poly-Cys-g-PDA@GPUF was not affected by pH. Khalilifard and Javadian [18] investigated the effect of magnetic superhydrophobic polyurethane sponge loaded with Fe_3_O_4_@oleic acid@graphene oxide as a high-performance adsorbing oil from water. The authors claimed that the modified PU sponge showed superhydrophobic properties, high performance in adsorbing organic solvents and oils, can be used 15 times without loss of adsorption capacity, and can be used to separate oil from water in static and dynamic states.

Oil separation can also be treated by electroflotation [19,20,21], which consists of the electrochemical generation of species within a solution by the application of electric current using sacrificial electrodes such as aluminium or iron. The removal rate of the contaminants depends on several experimental parameters, such as the initial concentration of the reagent, the electric current density, the arrangement of the electrodes, and others [19,20,21]. The combination of the electroflotation method with graphene-containing PU foams seems to be promising and effective as an oil separator.

Therefore, the objective of this work is to evaluate the performance of two-unit processes for the treatment of oil-contaminated water, namely a fixed-bed sorption column packed with polyurethane foam/graphene nanoplatelets and an electroflotation process. To achieve this objective, a method was proposed to improve the oil sorption capacity of flexible polyurethane foam by formulating a polyurethane foam/graphene nanoplatelet composite with and without the use of a dispersant. In addition, a bench-scale continuous electroflotation reactor was designed, also for the treatment of oil-contaminated wastewater. The combined use of these processes in the same treatment system is promising.

## 2. Materials and Methods

### 2.1. Materials

The following materials were used for the foam production: polyol (Voranol TM 3010) (styrene acrylonitrile (5 to 10%) and polyol polyester (85 to 95%) copolymer and hydroxyl index of 49 to 56 mg KOH·g^−1^, while colour and liquid) and toluene diisocyanate (TDI) (Voranate^TM^ T-80 TDI) were gently donated by Dow Brasil Sudeste Industrial Ltda., São Paulo, SP, Brazil. (lox acidity, 80/20 composition, with 80% of its isomerism in the form of toluene 2,4-diisocyanate and toluene 2,6-diisocyanate, molecular mass of 174 g·mol^−1^ and density of 1220 kg·m^−3^), triethylenediamine Dabco^®^ 2033 Catalyst by Air Products Brasil Ltda. (São Paulo, SP, Brazil). (dynamic viscosity of 0.154 kg·m^−1^·s^−1^, specific gravity of 990 kg·m^−3^ and hydroxyl number (OH) of 799 mg KOH·g^−1^), tin octoate (II) Kosmos^®^ 29 by Evonik Industries (São Paulo, SP, Brazil) (viscosity of 0.27–0.43 Pa·s and density of 1250 kg·m^−3^), urethane grade methylene chloride (low boiling point (39.9 °C), density of 1220 kg·m^−3^, molecular mass of 84.4 g·mol^−1^ and viscosity of 0.00041 Pa·s) supplied by Labsynth Produtos (Diadema, SP, Brazil), surfactant Niax Silicon L-95 (light yellow colour liquid, and density of 1027 kg·m^−3^) by Momentive Performance Materials Inc. (Itatiba, SP, Brazil), 1-methyl-2-pyrrolidone PA ACS (colourless liquid and pH of 7.7–8) by Êxodo Científica Química Fina Indústria e Comércio Ltda. (Sumaré, SP, Brazil), graphene nanoplatelet (6–8 nm thick × 5 microns wide) by Strem Chemicals Inc. (Newbutyport, MA, USA).

For sorption and electroflotation experiments, mineral motor oil Evora Super 20W-50 (Erechim, RS, Brazil) (density of 0.8711 g·cm^−3^ at 20 °C, kinematic viscosity at 40 °C of 147.2 cSt, flash point of 220 °C, and pour point of −27 °C), anionic surfactant sodium lauryl sulphate (dodecyl sulphate) ≥ 90% (C_12_H_25_NaO_4_S), diesel oil (diesel S500) (kinematic viscosity at 40 °C from 2 to 5 cSt, density at 20 °C from 0.815 to 0.865, and flash point of 38 °C), and sodium chloride (NaCl) were used.

### 2.2. Methods

**Figure 1:**
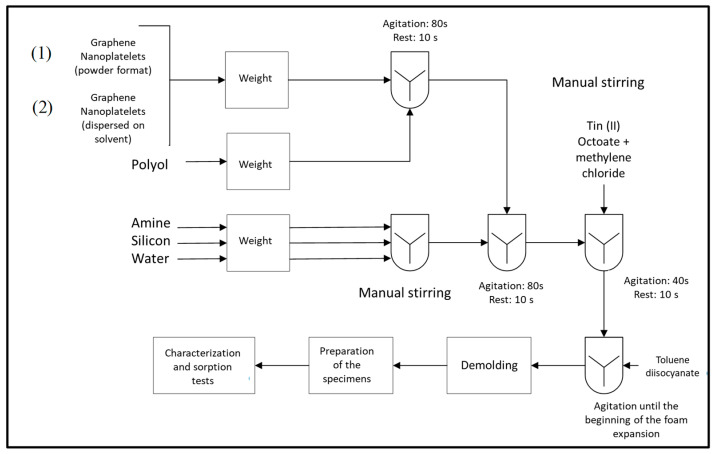
Schematic representation of the polyurethane/graphene nanoplatelet foam composites production.

The foams were produced using a flexible polyurethane with a theoretical density of 8 kg·m^−3^ according to a previous study (named PU8) [12] using a free blowing method with mechanical stirring (Fisatom equipment model 715 (FISATOM, São Paulo, SP, Brazil), at 100 rpm speed). Graphene nanoplatelets (GNP) (0.5% and 1%) were incorporated within the foam formulation, using the polyol reagent as a base. Two distinct procedures were applied: (i) GNP (powder format) was directly incorporated and mixed into the polyol, and (ii) GNP was previously dispersed in 24 g of 1-methyl-2-pyrrolidone by sonication (Hielscher UP400S, Hielscher Ultrasonics GmbH., Teltow, Germany) for 30 min, with an amplitude of 55% and a cycle of 0.5, prior to being incorporated into polyol. It produced four different PU foams, namely PUGN0.5 and PUGN1, with the incorporation of 0.5% and 1% GNP in powder format, and PUGNMP0.5 and PUGNMP1, with the incorporation of 0.5% and 1% GNP dispersed in 1-methyl-2-pyrrolidone, respectively. To compare, a PU foam without fillers was produced. Figure 1 presents the schematic representation of the polyurethane foam production.

The oil-in-water emulsion was produced by adding 100 mg·L^−1^ of mineral oil 20W-50 and 20 mg·L^−1^ of anionic surfactant in distilled water [22]. All reagents were added to a vessel and mechanically stirred for 30 min (Fisatom equipment model 715, at 1000 rpm speed, FISATOM, São Paulo, SP, Brazil) using a Cowles mixing propeller (50 mm diameter).

#### 2.2.1. Foam Characterisation

The specific gravity was determined according to ASTM D3574-11(A) [23].

The morphology was determined using field emission scanning electron microscopy (FESEM) (Tescan equipment (TESCAN, São Bernardo do Campo, SP, Brazil), model Mira 3, voltage of 15 kV, SEM HV of 10 KV, SEM magnifications of 50× and 5k×) on samples previously coated with gold.

The compression resistance was performed on an EMIC model DL 2000 (Porto Alegre, RS, Brazil) at 50 mm·min^−1^ (according to ASTM D3574-11(C) [23]), while the compression set was performed according to ASTM D395-16(B) [24] at two distinct temperatures: 23 and 70 ± 2 °C. 

The dynamic mechanical thermal analysis (DMTA) was determined using a TA instrument, model Q800 (TA Instruments, Newcastle, DE, USA), using cylindrical specimens of 40 mm diameter and 11 mm height on a compression clamp. The tests were carried out in non-isothermal conditions, from −60 °C to 100 °C at a heating rate of 3 °C·min^−1^, with a deformation set at 0.05% and a 1 Hz frequency.

The hydrophobic and sorption behaviour was determined by the contact angle and sorption capacity of oil in a static system and homogeneous medium, according to ASTM F726-17 [25].

#### 2.2.2. Sorption Test

The apparatus consists of a centrifugal pump (12 V, 800 L·h^−1^), a Fisatom mixer, model 715 (FISATOM, São Paulo, SP, Brazil) (1000 rpm) with a Cowles-type mixing disc dispersing propeller (50 mm diameter), and a polycarbonate column (260 mm height and 40 mm diameter). In the fixed bed column sorption system, the synthetic effluent was pumped to the system, and the output flow was adjusted to 40 mL·min^−1^. The foams were cut into polyhedra with a size of 5–10 mm.

#### 2.2.3. Electroflotation Treatment

**Figure 2:**
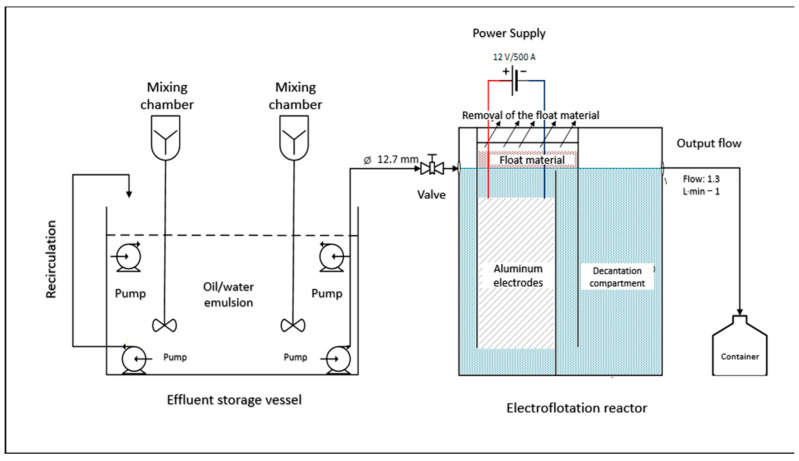
Schematic representation of the electroflotation apparatus.

**Figure 3:**
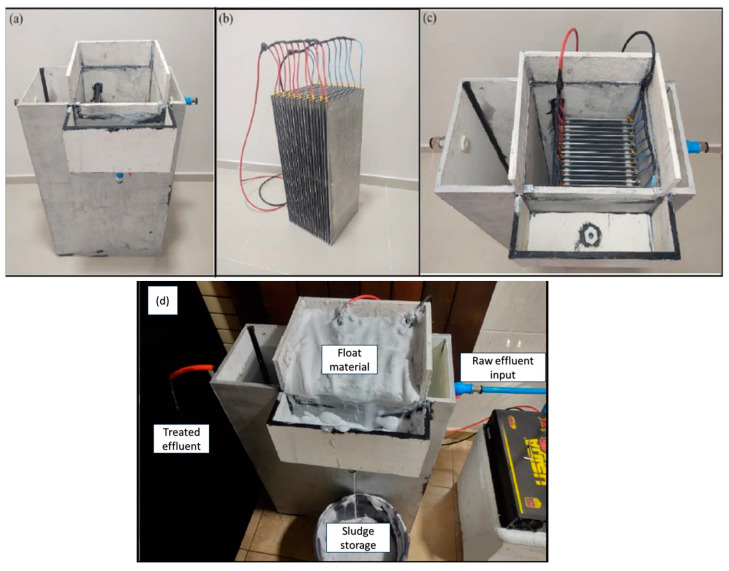
(**a**) Electroflotation storage vessel. (**b**) honeycomb-like aluminium electrodes; (**c**) electroflotation reactor; and (**d**) electroflotation reactor operating.

The apparatus for the oily contaminated effluent is represented in Figure 2 and Figure 3. The equipment is composed of a centrifugal pump (12 V, 800 L·h^−1^) (USINA PRO, Porto Alegre, RS, Brazil), an electrolytic reservoir (100 L), honeycomb-like aluminium electrodes composed of 20 rectangular sheets (235 × 625 × 2.5 mm) with a 10 mm gap between each electrode (30 L), an electrical connector in a monopolar arrangement in parallel, a polyethylene box (180 L), valves, connectors, and others.

The tests were performed on a reactor designed for continuous operation. The electrical voltage of the source was set at 12 V, and the inlet flow into the reactor was adjusted to maintain a hydraulic detention time of 30 min inside the electrode assembly. It was conducted 3 electroflotation experiments, according to Table 1.

The analyses of the raw effluent and of the treated one were conducted on duplicates, according to Standard Methods for Examination of Water and Wastewater [26]. From the results were estimated the contaminants removal total (oil, fats and anionic surfactants), as demonstrated in Equation (1):

(1) $$\%\;=\frac{C_{\mathrm{EB}}\;-\;C_{\mathrm{ET}}}{C_{\mathrm{EB}}}\cdot \;100\;$$

where $C_{\mathrm{EB}}$ is the concentration of the pollutant presented in the raw effluent and $C_{\mathrm{ET}}$ is the concentration of the pollutant presented in the treated effluent.

## 3. Results and Discussion

The density of the foams is represented in Table 2.

The density of PU8 increased by 14.13% compared to the theoretical value estimated at 8 kg·m^−3^ due to the variation in reagent measurements and times in the stages of mixture development and stirring. Fenner et al. [12] obtained a value of 10.2 ± 0.6 kg·m^−3^ using the same procedure. A slight increase was observed in comparison with the foams containing graphene nanoplatelets. The use of solvent did not significantly alter the values obtained. Piszczyk et al. [27] found an increase from 114.4 kg·m^−3^ for pure PU to 122.6 kg·m^−3^ for 0.75% graphene oxide. Li et al. [28] claimed an increase up to 1.5% oxide graphene, after which the values decreased. All results can be related to the opposite effect of nanoparticle incorporation in nucleation and cell growth. The nanoparticles act as nucleation sites, increasing the number of bubbles produced as the filler content increases. On the other hand, the viscosity is significantly increased, making it more difficult for the foam to grow.

**Figure 4:**
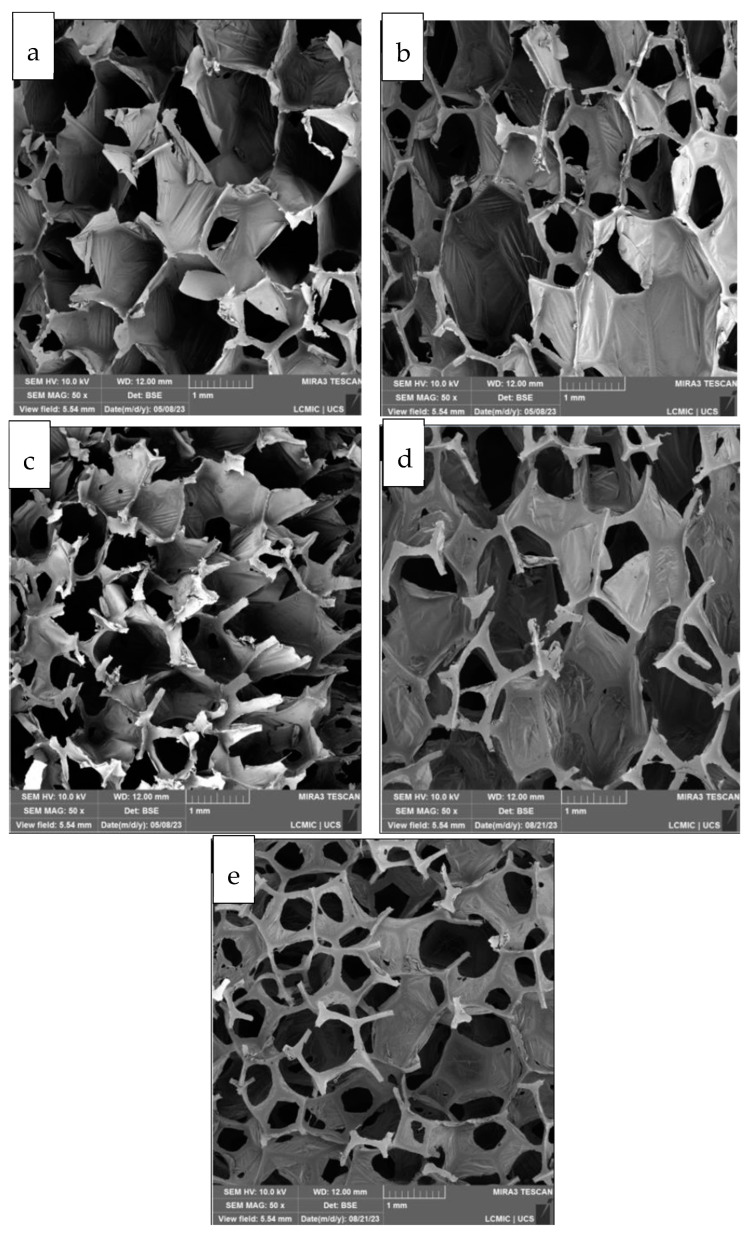
Morphology of the foams: (**a**) PU8; (**b**) PUGN0.5; (**c**) PUGN1; (**d**) PUGNMP0.5; (**e**) PUGNMP1.

Figure 4 shows the morphology of the investigated foams. In general, an interconnected 3D porous structure with both open and closed cells was observed. This is interesting for the sorption process, as the open cells promote interconnection with neighbouring cells in the inner core of the foam, while the closed cell provides a higher contact surface with the effluent and also makes the sorbed oil more difficult. Porosity also increased with graphene, hindering intercellular bonding due to a destabilising effect of graphene through cell edge retraction mechanisms. The solvent-containing foams promoted a higher number of pores compared to the solvent-free foam with the same graphene content. PUGNMP1 (Figure 4e) showed the higher number of pores. Finally, a slight tendency to decrease the pore size is observed with increasing graphene content (from 0.5% to 1%).

**Figure 5:**
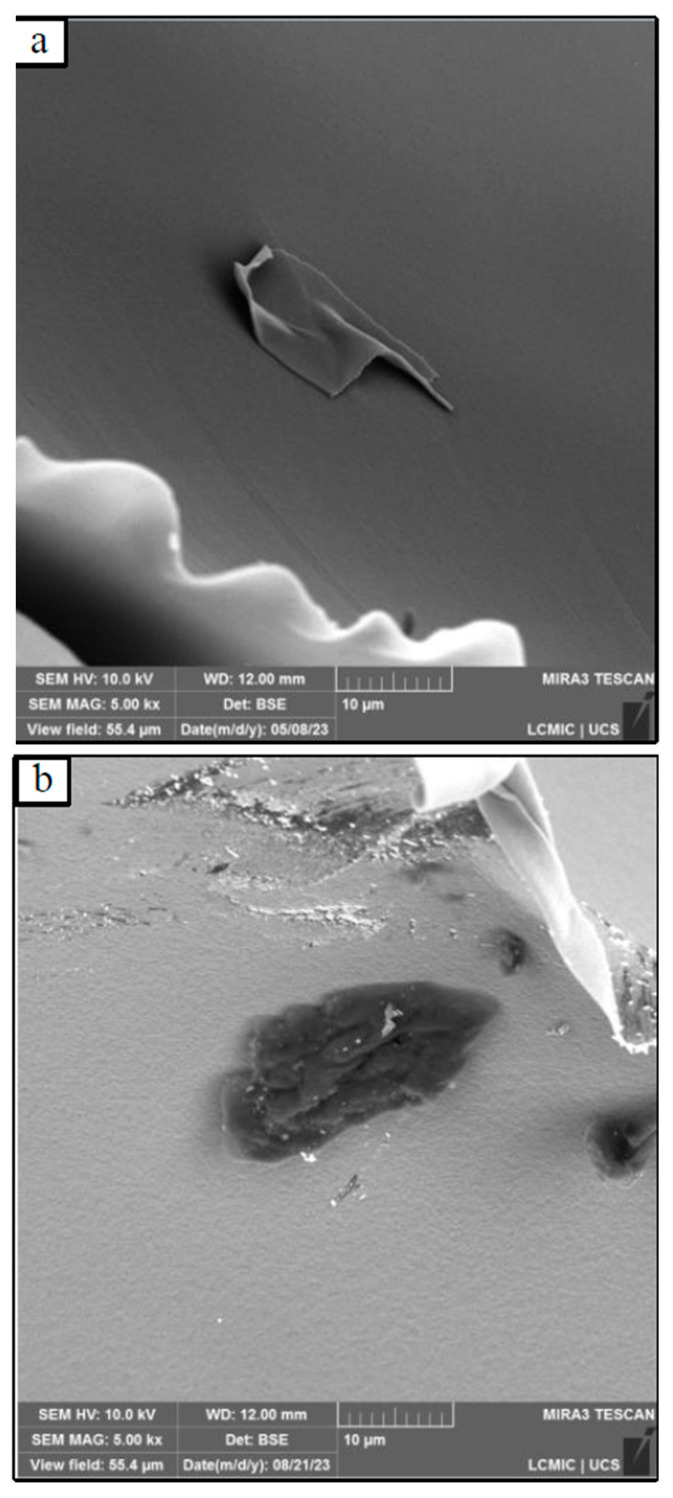
Microtopography of the surface for (**a**) PU8ande and (**b**) PUGNMP0.5, with a magnification of 5000 times fold.

The incorporation of graphene on 1-methyl-2-pyrrolidone promoted an increase in the rugosity of the foams, as observed in Figure 5 for two different foams. This fact suggests that 1-methyl-2-pyrrolidone not only acts as a dispersant for graphene nanoplatelets but also participates in the chemical reactions of PU foam formation. Kong et al. [29] used 1-methyl-2-pyrrolidone as a graphene dispersing solvent for the formulation of graphene-modified PU foams and also concluded that, in addition to the dispersing function, 1-methyl-2-pyrrolidone can act as a weak amine catalyst for foam polymerisation reactions.

**Figure 6:**
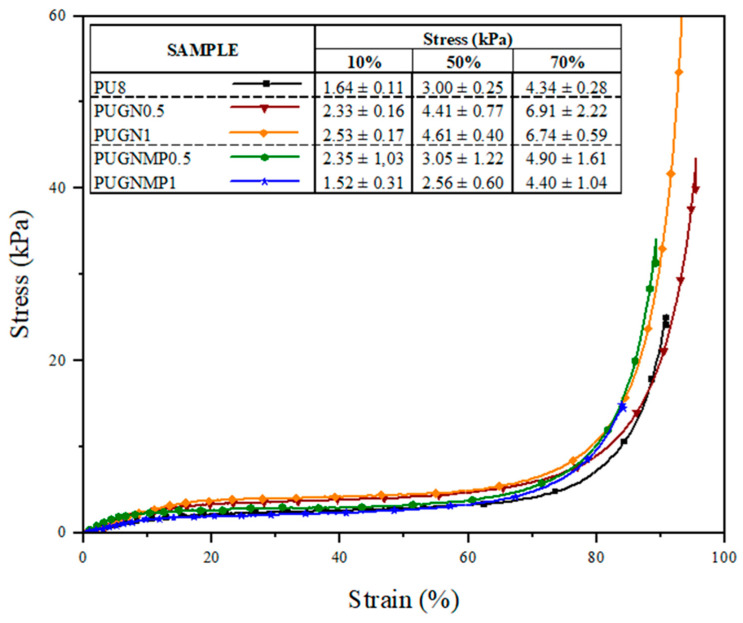
Stress versus strain curves for the foams under compression and for the foams produced.

The compressive mechanical behaviour of polymeric foams is strongly dependent on the type of foam (rigid or flexible), the cell type (open or closed), the density of the foam (void content), the size and density of the cells, among other factors [30]. Figure 6 shows the compression resistance of the foams produced. By analysing the curves obtained by compressing the foams, with deformation of up to 80% of the initial volume, it was observed that all the samples showed the typical deformation behaviour of a polymeric foam, with three well-defined phases: deformation in the elastic region, plateau, and densification [31]. The addition of carbon structures to the polymer matrix increases the compressive strength of the foams. Regarding the use of 1-methyl-2-pyrrolidone as a dispersant for the carbon structures, good compressive strength results were observed at a concentration of 0.5% graphene nanoplatelets. As the concentration increased to 1%, there was a decrease in compressive strength, even lower than that of the PU8 sample. It is suggested that this reduction in the compressive strength of the PUGNMP1 sample, which is even lower than that of the PU8 sample, is related to the greater number of pores in the foam cells as evidenced by the morphological analysis (Figure 4e). Furthermore, according to Li et al. [28], at a high filler content, agglomerates can become stress concentration points in the system, reducing their reinforcing effect and mechanical performance.

**Figure 7:**
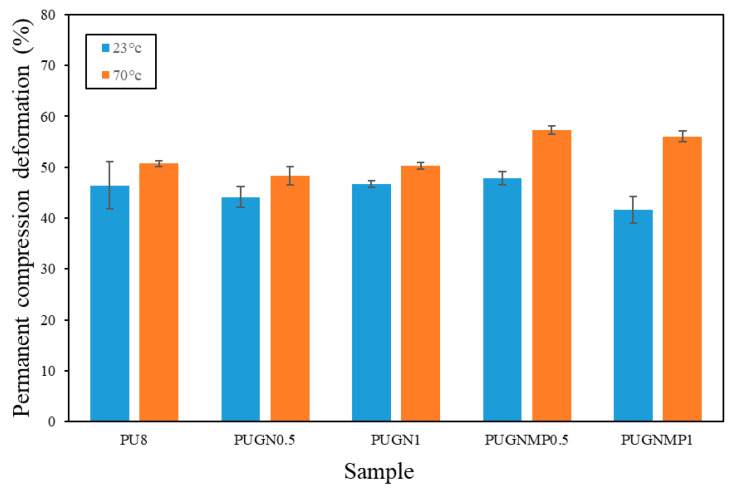
Permanent compression deformation of the studied samples at 23 and 70 °C.

Figure 7 shows the permanent compression deformation (PCD) of the foams produced. Graphene did not lead to a reduction in PCD compared to the unreinforced polyurethane foam (PU8) at 23 °C. On average, the foams showed a PCD of 45.3%, with the lowest value found being 41.6% for PUGNMP1 and the highest being 47.9% for PUGNMP0.5. On the other hand, the samples where 1-methyl-2-pyrrolidone was used as a filler dispersant had a negative effect on the mechanical resistance at 70 °C. On average, the samples without the dispersant deformed 49.3%, while those with dispersant deformed 56.7%. It is suggested that this behaviour may be related to two factors. Firstly, the greater porosity of the cells suggests that more porous cells will eventually be compressed and destroyed more easily than foams with closed cells.

**Figure 8:**
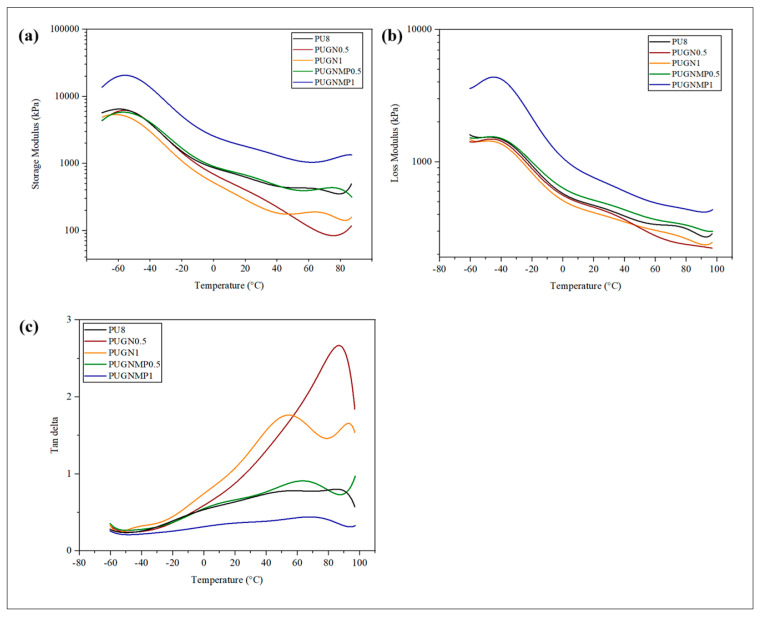
Dynamic mechanical thermal curves for the PU foam and the nanocomposites produced: (**a**) storage modulus, (**b**) loss modulus, and (**c**) tan δ.

Figure 8 shows the storage modulus, loss modulus, and tan delta curves obtained by DMTA for the foams produced. The storage modulus (E′) is directly related to the stiffness of the sample and determines the elastic energy stored by the material. The foams in which graphene was incorporated directly into the polyol obtained a lower mechanical performance compared to the PU8 foam, with a consequent reduction in the stiffness of the material when the load increased from 0.5% to 1%. On the other hand, the previous dispersion of graphene in 1-methyl-2-pyrrolidone, prior to its addition to the polyol, improved the mechanical performance when the load increased from 0.5% to 1%, promoting an increase in the stiffness of the material. This increase can be better observed by comparing the values obtained at a temperature of 25 °C. The E′ values, from the lowest to the highest, were 319 kPa for PUGN1, 472 kPa for PUGN0.5, 682 kPa for PU8, 737 kPa for PUGNMP0.5, and 1943 kPa for PUGNMP1, confirming the fact that the use of the dispersing solvent tends to distribute the load more uniformly in the polymer matrix.

The loss modulus (E″) is directly proportional to the energy dissipated or lost in the form of heat. E″ is an irreversible measurement and corresponds to the energy lost through the viscous response of the material. Figure 8b shows the E″ values of the samples and the viscous response of the foams with the addition of fillers, which shows a similar behaviour to that of the storage modulus.

The tan δ (Figure 8c) allows the evaluation of the difference between the elastic and viscous components of the material, and in a typical DMTA curve for PU foam, two peaks can be seen, at temperatures between −120 and −30 °C, related to the energy loss associated with the flexible segments of the PU structure, and in the temperature range from 0 to 90 °C, the energy loss associated with the rigid segments of the PU structure [32,33]. It was not possible to verify the energy loss peaks for the flexible PU segments, possibly because this relaxation event started and peaked at temperatures below −60 °C. On the other hand, the energy loss peaks for the rigid PU segments can be verified in the temperature range from 0 to 100 °C. The peak height is related to the energy loss when a main transition occurs [32]. PUGNMP1 has the lower peak height, while PUGN0.5 has the highest, indicating that the former has a higher energy capacity at this temperature compared to the latter.

The hydrophobicity of foams was evaluated by measuring the water contact angle (WAC) (Table 3).

The water contact angles obtained for all samples tested were greater than 90° at both t = 0 and t = 5 min, indicating the hydrophobicity of the foams. At t = 0, the lower contact angle measured was 107.7° ± 4.1 for PUGNMP0.5 and the higher contact angle was 117.8° ± 3.5 for PUGN1. The contact angle of water with the foam surface is related to the chemical composition and surface roughness of the material [34]. In a PU foam, the polar groups organic ether, carbamate, and amide are responsible for imparting the hydrophilic properties that allow the PU foam to absorb both water and oil and also to impart a smaller contact angle with water. In contrast, the incorporation of graphene nanoplatelets into PU foams, which are non-polar, electrically neutral substances with low surface energy, tends to cancel out some of the surface energy provided by the polar groups. However, as the nanofillers in the composite foams were incorporated into the polymer matrix, they did not significantly alter the surface energy of the foams to give contact angles with water above 150° [4,5,7].

**Figure 9:**
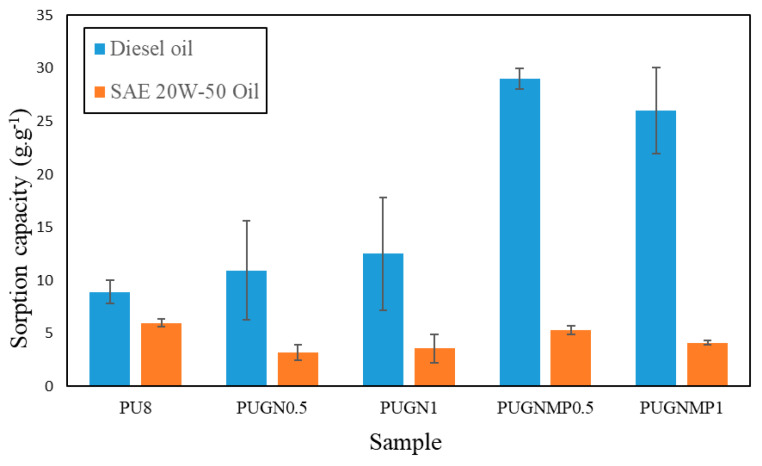
Sorption capacity of the foams produced tested on diesel oil and SAE 20W-50 oil.

Figure 9 shows the results of the static sorption capacity in a homogeneous medium of the produced foams in diesel oil and SAE 20W-50 oil.

The diesel oil sorption capacity increased with the incorporation of graphene nano-platelets in the PU foams. The addition of 1-methyl-2-pyrrolidone improved the sorption capacity compared to the samples without dispersant. PUGNMP0.5 exhibited the highest sorption capacity for diesel oil of 29 g·g^−1^, which is 3.3 times higher than the sorption capacity observed for PU8. It is suggested that this increase in sorption capacity for diesel oil is related to the increase in porosity and roughness of the microtopography as shown in the SEM/FEG micrographs (Figure 4 and Figure 5). On the other hand, the sorption capacity of SAE 20W-50 oil presented much lower values compared to the sorption capacity of diesel oil, in the range of 3 to 6 g·g^−1^, indicating that the higher viscosity of mineral oil made it difficult for the fluid to enter and conduct into the foams, concentrating the oil sorption process on the surface of the sorbent material, which did not occur with diesel oil, which has a lower viscosity compared to mineral oil. In addition, PU8 obtained the highest sorption capacity of SAE 20W-50 oil, possibly due to its structure, which is mainly composed of closed cells, as shown in Figure 4a, which provides a larger external contact surface, making it difficult to desorb the more viscous oil [4,7].

**Figure 10:**
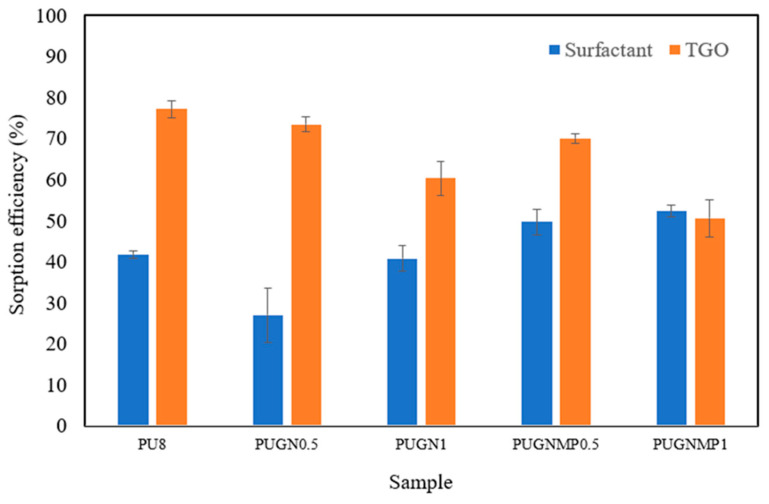
Sorption efficiency of surfactant and TGO for the foams produced.

The results of sorption tests performed in a flow fixed bed column are presented in Figure 10.

The foams produced were able to sorb the oil chemically emulsified by anionic surfactants, reducing the oil concentration present in the raw effluent by up to 77.2%. PU8, followed by PUGN0.5 and PUGNMP0.5, were the foams that showed the best emulsified oil removal efficiencies, possibly due to the fact that they have a structure composed mainly of closed cells (Figure 4a,b,d), which causes the outer surface of the foams to have a larger contact area with the oil. On the other hand, PUGN1 and PUGNMP1, which have a structure composed mainly of open cells (Figure 4c,e), had a lower sorption efficiency for SAE 20W-50 oil than the others. These results can be explained by the high viscosity of the mineral oil, which prevented the oil from diffusing into the foam, causing the adsorption phenomenon to occur only on the outer surface of the foams, as demonstrated in the sorption capacity tests in a static system and homogeneous medium [8,9]. It is also important to highlight the negative effect of increasing the filler content in the composite foams from 0.5% to 1% on the sorption capacity of emulsified oil. This behaviour can be explained by the increase in the specific density of the foams as the solid filler content increases and the consequent reduction in cell size, resulting in a loss of sorption capacity for heavier oils. However, it should be noted that this behaviour was not observed for light oils, such as diesel oil, as shown by the sorption tests in a static homogeneous medium. Regarding the sorption capacity of anionic surfactants, the maximum sorption efficiency achieved after passing through the fixed bed column was 52.4% for PUGNMP1. The low sorption capacity of surfactants by foams can be explained by the dual properties of hydrophobicity and hydrophilicity present in the molecules of anionic surfactants. While the non-polar part of the molecule is mainly adsorbed on the surface of the sorbent, the polar end makes it difficult to penetrate into the interior of the foam. Another factor to consider is that as the oil is sorbed by the foam, the droplets coalesce, releasing more surfactant molecules that end up free in the medium [4,8]. Competition to occupy the sorption surface must also be considered, as the surfactant molecules end up competing with the oil to occupy this space, and because the oil is virtually non-polar, it ends up having an advantage. In the case of anionic surfactant sorption, foams with greater porosity and intercellular connectivity (PUGN1, PUGNMP0.5, and PUGNMP1) achieved greater sorption efficiency compared to PU8 and PUGN0.5 foams, possibly due to the fact that surfactant molecules and micelles not adhering to the oil droplets could more easily diffuse into the interior of the foam and adsorb on the internal surface. The electroflotation reactor operated as designed, producing a quantity of floatable material that increased as the electric current increased. The presence of dark spots in the floated sludge was visually observed, indicating the presence of oils and fats in the sludge. The presence of small flakes of aluminium hydroxides and polyhydroxides in suspension was also observed in the last chamber of the reactor, suggesting that this chamber was undersized or that an additional filtration system is required to separate such material, such as the use of polyurethane sorbent foams [4,5,6,7,8,9].

**Figure 11:**
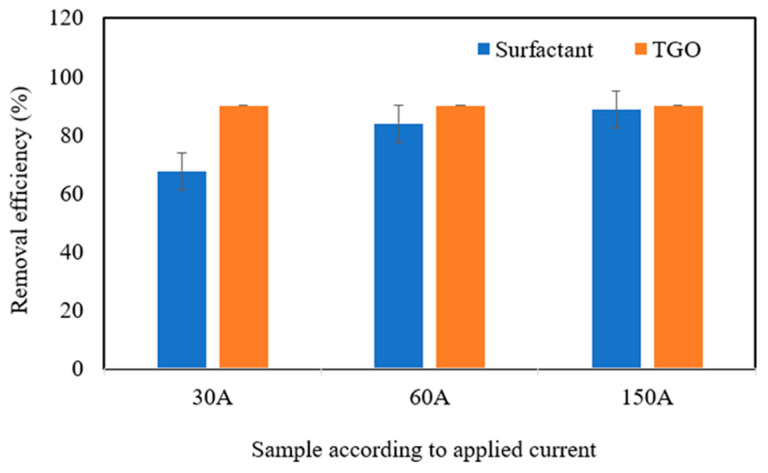
Removal efficiency of surfactant and TGO under different electrical currents.

Figure 11 shows the results of the total oil and grease removal efficiency of the continuous flow electroflotation treatment system.

The results showed that the ascending continuous flow electroflotation system using a set of vertical electrodes achieved excellent performance in removing total oils and fats at the three electric currents applied. The removal efficiency was between 90 and 100%, since the quantification limit for the analysis of total oils and fats is 10 mg·L^−1^. The efficient separation result of the oil/water chemical emulsion can be explained by the process of electrochemical destabilisation of the oil droplets chemically emulsified by anionic surfactants. According to this mechanism, the oil droplets were stabilised by anionic surfactants, which have a high spatial effect and electrostatic attraction, present a distribution of negative charges on the surface, and combine with the metallic hydroxyl compounds, resulting in charge neutralisation, oil droplet destabilisation, and flocculation. In addition, the electric field also had a demulsifying effect, polarising and deforming the small oil droplets, facilitating coalescence, flotation, and adsorption to form flocs [35]. Since the oil removal efficiency was the same regardless of the applied electric current, it is suggested that the designed electroflotation system was able to remove the emulsified oil at the lowest applied current density of 10 A·m^−2^. Contrary to the results for total oil and grease removal efficiency, in the case of anionic surfactants, the intensity of the electric current applied to the electrode set influenced the removal of these contaminants. As the electrical current increased, there was an increase in surfactant removal efficiency. However, it was observed that the increase in surfactant removal was not proportional to the increase in electrical current, as a 100% increase in electrical current, from 30 to 60 A, promoted a 24% increase in removal efficiency, whereas a 300% increase in current, from 60 to 150 A, promoted only a 5.6% increase in surfactant removal. A possible explanation for the behaviour described above could be the effect of the concentration of free surfactants in the medium. When the concentration of surfactants is high, the probability of collision of the adsorbent flakes is greater. However, as the surfactants are adsorbed, their concentration in the medium decreases, reducing the probability of collision with the adsorbent flakes. Although the electroflotation treatment achieved good surfactant removal results, the energy consumption associated with the increase in current from 60 A to 150 A does not allow for the small increase in surfactant removal efficiency. It would be interesting for future work to evaluate the effect of hydraulic retention time and surfactant concentration [32,34,35].

It should also be noted that as the current increases, there is a proportional increase in the dissolution of the aluminium electrodes and the formation of metal cations, which promotes greater production of floated sludge, increasing the cost of the electroflotation process. In addition, the production of more coagulant by increasing the electrical current without an equivalent amount of contaminants in the medium results in a higher concentration of dissolved aluminium in the treated effluent, which may violate standards for disposal or reuse of the effluent. A comparative analysis has highlighted some advantages and disadvantages of the sorption column and the electroflotation reactor and the feasibility of using the two processes together (Table 4).

Taking into account the advantages and disadvantages of both systems discussed in this work, it is suggested that the combined use of the systems may be an interesting alternative for the treatment of effluents contaminated with oil emulsified by anionic surfactants. The use of a sorption column upstream of the electroflotation reactor could separate some of the oil and allow it to be recycled. It could also reduce the concentration of oil entering the electroflotation reactor, which may require the use of a lower electrical current, resulting in less floated sludge. The use of another sorption column after the electroflotation reactor would help to retain any aluminium flakes from the electrolytic treatment and would also act as an additional safety system in the event of a problem with the electroflotation reactor.

Oil/water emulsions are heterogeneous mixtures in which small oil droplets, generally less than 20 microns in diameter, are dispersed in water. These droplets are stabilised by natural or added surfactants (such as detergents), which makes oil-water separation particularly difficult. The treatment of emulsions therefore requires different strategies and specific technologies. The main techniques used to remove oil from water include coagulation-flocculation, membrane filtration, centrifugal separation, bioremediation, sorption, and electroflotation. Both sorption with treated PU foams and electroflotation are viable and efficient techniques for treating water contaminated with oil in emulsion. Each has specific advantages depending on the desired application: sorption is characterised by its simplicity and low cost, while electroflotation offers greater automation and performance in stable emulsions. The integration of both techniques in hybrid systems is a promising strategy to increase efficiency and operational flexibility, low cost and scalability of the technology in different industrial contexts. The choice of the ideal technique depends on several factors, such as the type of water (fresh or saline), the type and stability of the emulsion, the amount of oil, the volume of water to be treated, the availability of infrastructure and energy, and legal and environmental requirements. In many cases, combinations of techniques (e.g., sorption pretreatment followed by electroflotation) can offer efficient combinations in terms of cost/benefit.

The electroflotation reactor, designed to operate at 150 A and 12 V, has a power of 1800 W and is capable of treating oily effluent at a flow rate of 60 L/h. The current of 150 A can be considered high for the process because, in addition to the high energy consumption, the increase in current leads to increased dissolution of the aluminium electrodes and the formation of metal cations, increased sludge production, increased concentration of residual aluminium in the treated effluent, and an increase in the pH of the treated effluent. Considering that the sorption column used in the tests, without packing, has a useful volume of approximately 330 cm^3^, the flow rate of 40 mL cannot be considered low. For example, if a sorption column with a usable volume of 33 L were designed on a scale of 100×, the flow rate in the column would be 4 L/min or 240 L/h, four times higher than in the electroflotation reactor.

## 4. Conclusions

The methodology used to formulate flexible PU foams incorporating solid or dispersed graphene nanoplatelets in 1-methyl-2-pyrrolidone was efficient. The morphological analyses showed that the prepared foams exhibited a 3D integrated porous network structure containing open and closed cells. With the addition of fillers to the PU foam matrix, an increase in the number of pores was observed, favouring intercellular interconnection. In addition, the use of 1-methyl-2-pyrrolidone as a filler dispersing solvent promoted the roughness of the microtopography of the surface of the foams. In general, graphene promotes an increase in the compressive strength of the composite foams, which helps to avoid deformation of the sorbent when used as a packing in sorption columns. The compression durability tests did not show that the addition of graphene nanoplatelets as reinforcement significantly improved this property. On the other hand, it was observed that in the test at a temperature of 70° C, the samples in which 1-methyl-2-pyrrolidone was used had a greater permanent deformation under compression than the samples in which the solvent was not used. The effect of using 1-methyl-2-pyrrolidone as a solvent to disperse the fillers was evaluated by DMTA analysis, which confirmed that the dispersant improved the mechanical performance by increasing the stiffness of the PU foam/graphene nanoplatelet composites. Contact angle analysis with water showed that the developed foams are all hydrophobic with values between 107.7° and 117.8°.

Furthermore, sorption tests in a static medium showed that the incorporation of fillers into PU foams promoted an increase in sorption capacity for less viscous oils such as diesel oil, which was more pronounced for foams in which the dispersing solvent was used, due to the greater porosity and roughness of the surface microtopography of the foams. The composite foams produced and used as packing in a fixed bed sorption column reduced the concentration of chemically emulsified oil by up to 77.2% and the concentration of anionic surfactants by up to 52.4%. PU8 had the highest sorption efficiency for oil, and PUGNMP1 had the highest sorption efficiency for anionic surfactants. The developed continuous electroflotation reactor showed a high separation capacity of chemically stabilised oil emulsions, above 90% in all tests performed, and separation of anionic surfactants of up to 88.6%, using an electric current of 150 A.

The effluent treatment methods developed in this study have the potential to be used in combination. The sorption column with PU foam could be used upstream of the electroflotation reactor to reduce the pollutant load and separate some of the emulsified oil for recycling and downstream of the reactor to retain small aluminium hydroxide flakes that have passed through the electroflotation reactor and also to act as an auxiliary safety device in case the efficiency of electroflotation is compromised by deviation of some control parameters. Activated carbon, although widely used in water treatment, has poor performance on emulsified oils, as it is not selective and tends to saturate quickly with other organic compounds present. Once saturated, the carbon is discarded and cannot be reused. Its use is more appropriate as a final step (polishing) in systems where the oil load has already been significantly reduced by other methods.

## Figures and Tables

**Table 1:** Operational parameters used in the electroflotation experiments.

Test	NaCl (g·L^−1^)	Electric Current (A)	Electric Current Density (A·m^−2^)
01	0.025	30	10
02	0.05	60	20
03	0.15	150	50

**Table 2:** Density of the different foams produced.

Specimen	Density (kg·m^−3^)
PU8	9.13 ± 0.12
PUGN0.5	11.57 ± 0.83
PUGN1	11.42 ± 1.55
PUGNMP0.5	10.93 ± 0.43
PUGNMP1	11.46 ± 1.41

**Table 3:** Contact angle of the polyurethane and the composites produced.

Sample	WCA (t = 0)	WCA (t = 5 min)
PU8	113.0° ± 4.6	107.0° ± 8.6
PUGN0.5	117.6° ± 5.3	114.3° ± 2.8
PUGN1	117.8° ± 3.5	110.2° ± 5.7
PUGNMP0.5	112.7° ± 3.91	105.9° ± 2.0
PUGNMP1	107.7° ± 4.1	103.7° ± 2.6

**Table 4:** Advantages and disadvantages of the sorption column and the electroflotation reactor methods.

Method	Advantages	Disadvantages
Sorption column	- Maximum efficiency of around 77% for sorb oil chemically emulsified by anionic surfactants - Maximum efficiency of around 52% for anionic surfactant sorption - Relatively low energy consumption, since it operates only by means of pumps - Possibility of collecting and recycling the sorbed oil by compressing the foams - It requires a small area for installation	- If used individually, it is not capable of meeting the effluent disposal standards established by Brazilian legislation - After being saturated by several uses, the PU foam needs to be replaced, and the waste generated is classified as hazardous, requiring proper final disposal
Electroflotation reactor	- High treatment efficiencies, practically 100% for total oils and greases and up to 88% for anionic surfactants - Automation is feasible - It can be designed with compact equipment and in a small area, compared to other single-unit effluent treatment processes - It can be designed with the use of solar energy, making its use in remote regions feasible	- Energy consumption can be high when compared to other treatment processes - Aluminium electrodes need to be replaced periodically - Additional treatment of the sludge generated by flotation is required - Denser aluminium flakes or those with a density close to that of water can be carried through the reactor, increasing the aluminium concentration of the treated effluent

## Data Availability

Data are contained within the article.
